# Supplementary material for: First Records of Possibly Human Pathogenic Rickettsia Species in Bat Ticks, Carios vespertilionis, in Sweden
Source: Microorganisms. 2023 Jan 31;11(2):357. doi: 10.3390/microorganisms11020357 (PMC9959223; doi:10.3390/microorganisms11020357)
Supplement: Supplementary file 1 [file microorganisms-11-00357-s001.zip › microorganisms-2081486-supplementary.pdf]

Supplementary File 1. Aligned rickettsial nucleotide sequences based on PCR-products.

The identifier line, which begins with '>', gives the name of the **microorganism_gene target_extra sample info**

>Rickettsia sp gltA (I)
ATCATTCATTAGTGAATGAAAGATTACACTATTTATTTCAAACCTTTTGTAGCTCTTCTCATCCTATGGCTATTAT
GCTTGCGGCTGTCGGTTCTCTTTCGGCATTTTATCCTGATTTATTGAATTTTAAGGAAGCAGATTACGAACTTAC
CGCTATTAGGATGATTGCTAAGATACCTACCATCGCTGCAATGTCTTATAAATATTCTATAGGACAACCGTTTAT
TTATCCTGATAATTCATTAGATTTTACCGAAAATTTTCTGCATATGATGTTTGCAACGCCTTGTACGAAATATAC
AGTAAATCCAATAATAAAAAATGCTCTTAATAAGATATTTATCCTACATGCCGATCATGAGCAGAATGCTTCTAC
TTCAACAGTCCGAATTGCCGGCTCATCCGGAGCTAACCCTTTTGCTTGTATTAGCACGGGTATTGCCTCACTTTG
GGGACCTGCTCACGGCGGGGCTAATGAAGCGGTAATAAATATGCTTAAAGAAATCGGTAGTTCTGAGTATATT
CCTAAATATATAGCTAAAGCTAAGGATAAAAATGATCCATTTAGATTAATGGGTTTTGGTCATCGTGTATATAA
AAACTATGACCCGCGTGCCGCAGTACTTAAAGAAACGTGCAAAGAAGTATTAAAGGAACTCGGGCAGCTAGA
CAACAATCCGCTCTTACAAATAGCAATAGAACTTGAAGCTATCGCTCTTAAAGATGAATATTTTATTGAGAGAA
AATTATATCCAAATGTTGATTTTTATTCGGGTATTATCTATAAAGCTATGGGTATACCGTC

>Rickettsia sp 17kDa
GCTCTTGCAACTTCTATGTTACAAGCCTGTAACGGTCCGGGCGGTATGAATAAACAAGGTACAGGAACACTTCT
TGGCGGTGCTGGCGGCGCATTACTTGGTTCTCAATTCGGTAAGGGCAAAGGACAGCTTGTTGGAGTAGGTGT
AGGTGCATTACTTGGAGCAGTTCTTGGTGGACAAATCGGTGCAGGTATGGATGAACAGGATAGAAGACTTGC
AGAGCTTACCTCACAGAGAGCTTTAGAAACAGCTCCTAGTGGTAGTAACGTAGAATGGCGTAATCCGGATAAC
GGCAATTACGGTTACGTAACACCTAATAAAACTTATAGAAATAGCACTGGTCAATATTGCCGTGAGTACACTCA
AACAGTTGTAATAGGCGGAAAACAACAAAAAGCATACGGTAATGCATGCCGCCAACCTGACGAACAATG

>Rickettsia sp ompB
GGACTTAGCTACTTAAAGTCTTCTGACGAAAACTACAAAGAAACCGGTACAACAGTTGCAAACAAGCAAGTTA
ACAGCAAATTTAGCGATAGAACCGATTTAATAGTAGGTGCTAAAGTAGCCGGCAGTACTATGAACATAACTGA
TCTTGCGGTATATCCGGAAGTTCACGCTTTTGTGGTTCACAAAGTAACCGGTAGATTATCTAAAACTCAGTCTG
TATTAGACGGACAAGTTACTCCGTGTATCA

>Rickettsia sp ompB nymph 191 Uppsala 2018
GGACTTAGCTACTTAAAGTCTTCTGACGAAAACTACAAAGAAACCGGTACAACAGTTGCAAACAAGCAAGTTA
ACAGCAAATTTAGCGATAGAACCGATTTAATAGTAGGTGCTAAAGTAGCCAGTACTATGAACATAACTGATCTT
GCGGTATATCCGGAAGTTCACGCTTTTGTGGTTCACAAAGTAACCGGTAGATTATCTAAAACTCAGTCTGTATT
AGACGGACAAGTTACTCCGTGTATCAGC

>Rickettsia sp ompB nymph 229 Uppsala 2018
AGGACTTAGCTACTTAAAGTCTTCTGACGAAAACTACAAAGAAACCGGTACAACAGTTGCAAACAAGCTAGTT
AACAGCAAATTTAGCGATAGAACCGATTTAATAGTAGGTGCTAAAGTAGCCGGCAGTACTATGAACATAACTG
ATCTTGCGGTATATCCGGAAGTTCACGCTTTTGTGGTTCACAAAGTAACCGGTAGATTATCTAAAACTCAGTCT
GTATTAGACGGACAAGTTACTCCGTGTATCAG

>Rickettsia sp. AvBat ompA
TCACCACCTCAACCGCAGCGATAATGCTGAGTAGTAGCGGGGCACTCGGTGTTGCTGCAGGTGTTATTGCTAC
TAATAATAATGCAGCATTTAGTGATAATGTTGGCAATAATAATTGGAATGAGATAACGGCTGCAGGGGTAGCT
AATGGTAATCCTGCTGGCGGTCCTCAAAACAATTGGGCATTTACTTACGGTGGTGATTATACTATCACTGCAGA
TGCAGCCGATCGTATTATTACGGCTATAAATGTTGCGGGTACTACTCCCGTAGGTCTAAATATTGCTCAAAATA

CCGTTGTTGGTTCGATTATAACGGGAGGTAACTTGTTGCCTGTTACTATTACTGCCGGCAAAAGCTTAACTTTA
AACGGTAATAATGCTGTTGCTG

>Rickettsia sp. AvBat gltA adult 179 Uppland 2018
TAATGAAGCGGTAATAAATATGCTTAAAGAAATCGGTAGTTCTGAGTATATTCCTAAATATATAGCTAAAGC
TAAGGATAAAAATGATCCATTTAGATTAATGGGTTTTGGTCATCGTGTATATAAAAACTATGACCCGCGTGCCG
CAGTACTTAAAGAAACGTGCAAAGAAGTATTAAAGGAACTCGGGCAGCTAGACAACAATCCGCTCTTACAAAT
AGCAATAGAACTTGAAGCTATCGCTCTTAAAGATGAATATTTTATTGAGAGAAAATTATATCCAAATGTTGATT
TTTATTCGGGTATTATCTATAAAGCTATGGGTATACCGTC

>Rickettsia sp. AvBat rrs
TTTACCGTGGTTGGCTGCCTCTTGCGTTAGCTCACCACCTTCAGGTAAAACCAACTCCCATGGCGTGACGGGCA
GTGTGTACAAGGCCCGAGAACGTATTCACCGCGGCATGCTGATCCGCGATTACTAGCGATTCCAACTTCATGCT
CTCGAGTTGCAGAGAGCAATCTGAACTGAGATATCTTTTAGGGATTTGCTCCACGTCGCCGTATTGCTTCCCTCT
GTAGATACCATTGTAGCACGCGTGTAGCCCAACCCGTAAGGGCCATGATGACTTGACATCGTCCCCACCTTCCT
CCGGCTTATCACCGGCAGTTTCCTTATAGTTCCCGGCATTACCCGCTGGCAAATAAGAATGAGGGTTGCGCTCG
TTGCGGGACTTAACCCAACATCTCACGACACGAGCTGACGACAGCCATGCAACACCTGTGTGTGACCCAGCCG
AACTGAAGGGATGCATCTCTGCTTCCCGCGACCACCATGTCAAGGGTTGGTAAGGTTTTTCGCGTATCATCGAA
TTAAACCGCATGCTCCACCGCTTGTGCGAGCCCCCGTCAATTCCTTTGAGTTTTAATCTTGCGACCGTACTCCCC
AGGCGGAGTGCTTAATGCGTTAGCTGCGAAACCGAGAGAAATTCTCCCGATATCTAGCACTCATCGTTTACGG
CGTGGACTACCAGGGTATCTAATCCTGTTTGCTCCCCACGCTTTCGTGCATCAGCGTCAGTTGTAGCCTAGATG
ACCGCCTTCGCCACCGGTGTTCCTCCTAATATCTAAGAATTTCACCTCTACACTAGGAATTCCATCATCCCCTACT
ACACTCTAGATTAGTAGTTTTGAAAGCAGTTCCGAGGTTAAGCCTCGGGCTTTCACTTCCAACTTACTAAACCAC
CTACGCACTCTTTACGCCCAGTAATTCCGAACAACGCTAGCCCCCTCCGTCTTACCGCGGCTGCTGGCACGGAG
TTAGCCGGGGCTTTTTCTGCAGGTACCGTCATTATCTTCCCTGCTAAAAGAACTTTACAACCCTAAGGCCTTCAT
CATTCACGCGGCATTGCTGGATCAGGCTTTCGCCCATTGTCCAATATTCCCCACTGCTGCCTCCCGTAGGAGTCT
GGGCCGTGTCTCAGTCCCAGTGTGGCTGATCATCCTCTCAGACCAGCTACAGGTCGTTGGCTTGGTAGGCCATT
ACCCTACCAACTACCTAATCTGACGCGGGCTCATCCATCAGCGATAAATCTTTCCTCCGAAGAGAATATACGGT
ATTAGCATTTATTTCTAAATGTTATTCCGTACTGATGGGGAGATTCCCACGTGTTACTCACCCGTTTGCTACTAA
TTTATTCAGAGCAAGCCC

>Rickettsia sp. AvBat rrs nymph 159 Uppland 2018
TCACCCCAGTCGCTAATTTTACCGTGGTTGGCTGCCTCTTGCGTTAGCTCACCACCTTCAGGTAAAACCAACTCC
CATGGCGTGACGGGCAGTGTGTACAAGGCCCGAGAACGTATTCACCGCGGCATGCTGATCCGCGATTACTAG
CGATTCCAACTTCATGCTCTCGAGTTGCAGAGAGCAATCTGAAGAGATATCTTTTAGGGATTTGCTCCACGTCG
CCGTATTGCTTCCCTCTGTAGATACCATTGTAGCACGCGTAGCCCAACCCGTAAGGGCCATGAACTTGACATCG
TCCCCACCTTCCTCCGGCTTATCACCGGCAGTTTCCTTATAGTTCCCGGCATTACCCGCTGGCAAATAAGAATGA
GGGTTGCGCTCGTTGCGGGACTTAACCCAACATCTCACGACACGAGCTGACGACAGCCATGCAACACCTGTGT
GTGACCCAGCCGAACTGAAGGGATGCACTCTGCTTTCCCGCGACCACCATGTCAAGGGTTGGTAAGGTTTTTC
GCGTATCATCGAATTAAACCGCATGCTCCACCGCTTGTGCGAGCCCCCGTCAATTCCTTTGAGTTTTAATCTTGC
GACCGTACTCCCCAGGCGGAGTGCTTAATGCGTTAGCTGCGAAACCGAGAGAAATTCTCCCGATATCTAGCAC
TCATCGTTTACGGCGTGGACTACCAGGGTATCTAATCCTGTTTGCTCCCCACGCTTTCGTGCATCAGCGTCAGTT
GTAGCCTAGATGACCGCCTTCGCCACCGGTGTTCCTCCTAATATCTAAGAATTTCACCTCTACACTAGGAATTCC
ATCATCCCCTACTACACTCTAGATTAGTAGTTTTGAAAGCAGTTCCGAGGTTAAGCCTCGGGCTTTCACTTCCAA
CTTACTAAACCACCTACGCACTCTTTACGCCCAGTAATTCCGAACAACGCTAGCCCCCTCCGTCTTACCGCGGCT
GCTGGCACGGAGTTAGCCGGGGCTTTTTCTGCAGGTACCGTCATTATCTTCCCTGCTAAAAGAACTTTACAACC
CTAAGGCCTTCATCATTCACGCGGCATTGCTGGATCAGGCTTTCGCCCATTGTCCAATATTCCCCACTGCTGCCT

CCCGTAGGAGTCTGGGCCGTGTCTCAGTCCCAGTGTGGCTGATCATCCTCTCAGACCAGCTACAGGTCGTTGG
CTTGGTAGGCCATTACCCTACCAACTACCTAATCTGACGCGGGCTCATCCATCAGCGATAAAT

>Rickettsia sp. AvBat rrs nymph 161 Uppland 2018
TTTCCGGGATCGGGCTTAAGGAAAGGATCGGACTTCCCCCAGTCGCTAATTTTACCGTGGTTGGCTGCCTCTTG
CGTTAGCTCACCACCTTCAGGTAAAACTAACTCCCATGGCGTGACGGGCAGTGTGTACAAGGCCCGAGAACGT
ATTCACCGCGGCATGCTGATCCGCGATTACTAGCGATTCCAACTTCATGCTCTCGAGTTGCAGAGAACAATCCG
AACTGAGATGTCTTTTAGGGATTTGCTCCACGTCGCCGTCTTGCTTCCCTCTGTAAACACCATTGTAGCACGCGT
GTAGCCCAACCCGTAAGGGCCATGATTACTTGACGTCGTCCCCACCTTCCTCCGGCTTATCACCGGCAGTTTTCT
TATAGTTCCCGGCATTACCCGCTGGCAAATAAGAATGAGGGTTGCGCTCGTTGCGGGACTTAACCCAACATCTC
ACGACACGAGCTGACGACAGCCATGCAACACCTGTGTGTGACCCAGCCGAACTGAAGAAAAGCATCTCTGCTA
TCCGCGACCACCATGTCAAGGGTTGGTAAGGTTTTTCGCGTATCATCGAATTAAACCGCATGCTCCACCGCTTG
TGCGAGCCCCCGTCAATTCCTTTGAGTTTTAATCTTGCGACCGTACTCCCCAGGCGGAGTGCTTAATGCGTTAG
CTGCGAAACCGAAAGAAAATCTTCCGATATCTAGCACTCATCGTTTACGGCATGGACTACCAGGGTATCTAATC
CTGTTTGCTCCCCACGCTTTCGTGCATCAGCGTCAGTTGTAGCCTAGATGACCGCCTTCGCCACCGGTGTTCCTC
CTAATATCTAAGAATTTCACCTCTACACTAGGAATTCCATCATCCCCTACTACACTCTAGATTAATAGTTTTGAAA
GCAATTCCGAGGTTAAGCCTCGGGCTTTCACTTCCAACTTACTAAACCACCTACGCACTCTTTACGCCCAGTAAT
TCCGAACAACGCTAGCCCCCTCCGTCTTACCGCGGCTGCTGGCACGGAGTTAGCCGGGGCTTTTTCTGCAAGTA
ACGTCATTATCTTCCTTGCTAAAAAAACTTTACAACCCTAAGGCCTTCATCCATTCACTCGCAATTGTGGGATCA
GGTTTTCGCCCATTGTCCAATATTCCCCACTGCTCCTTCCGGTAGGATTTGGGCCCGGTTTTCATTCCCAGGGGG
GTGGATCATCTTTTCAGCCCAGTACCAGATCGTGGGCTGGGAGAGCCATTCCCTCCCCAACACCTAATTCTGAC
GGGGGTTCATCCTTCAGCGAAAATTTTTTCTTCGGCAGAGAATATACGGTATTACATTTTATTTCAAAAGTTATT
CCGTACTAAGGGGTAGATTCCCAGGGGTTATTCCCCCGTTTTCACAAATTAACGGAGCAAGCCCCAAAAATCC
GTCGACTGG
